# Efficacy and Safety of Bevacizumab in the Treatment of Pterygium: An Updated Meta-Analysis of Randomized Controlled Trials

**DOI:** 10.1155/2018/4598173

**Published:** 2018-09-05

**Authors:** Yi Sun, Bowen Zhang, Xiuhua Jia, Shiqi Ling, Juan Deng

**Affiliations:** ^1^Department of Ophthalmology, Third Affiliated Hospital of Sun Yat-Sen University, Guangzhou 510630, China; ^2^Surgical Department, The First Affiliated Hospital of Guangzhou Medical University, Guangzhou 510120, China

## Abstract

**Purpose:**

Studies investigating efficacy and safety of bevacizumab in pterygium have increased and reported controversial results. Thus, we updated this meta-analysis to clarify the issue.

**Methods:**

Studies were selected through search of the databases Embase, PubMed, Web of Science, and the Cochrane Central Register of Controlled Trials (CENTRAL) from their inception up until June 2017. The pooled risk ratio (RR) and 95% confidence interval (CI) were calculated for recurrence and complication rates by using random effects model.

**Results:**

1045 eyes in 18 randomized controlled trials (RCTs) enrolled. Overall, the pooled estimate showed a statistically significant effect of bevacizumab on the reduction of recurrence (RR 0.74, 95% CI 0.56–0.97, *P*=0.03). Subgroup analyses presented significant results beneficial to bevacizumab (primary pterygium group, RR 0.53, 95% CI 0.33–0.83, *P*=0.006; conjunctival autograft group, RR 0.48, 95% CI 0.25–0.91, *P*=0.02; and follow-up longer than 12 months group, RR 0.36, 95% CI 0.13–0.99, *P*=0.05). No statistically significant difference was observed in complication rates.

**Conclusions:**

Application of bevacizumab showed a statistically significant decrease in recurrence rate following removal of primary pterygia, or in cases with conjunctival autograft, or with follow-up longer than 12 months, while complications were not increased.

## 1. Introduction

Pterygium is one of the most common ocular surface diseases, which is characterized by the fibrovascular conjunctiva tissue proceeding from the bulbar conjunctiva towards the cornea. It limits eye movements and causes dry eye, irritation, foreign body sensation, and even decrease of visual acuity [1]. The primary treatment for pterygium is surgery, and the major problem of the treatment is the high recurrence rate, varying between 38% and 88% in bare sclera, 5%–30% in conjunctival autograft, and 0%–15% in limbal conjunctival autograft [2]. Many adjuvant therapies have been developed to reduce recurrence including mitomycin C [3, 4], 5-FU [5–7], and radiotherapy [8, 9].

In 2001, expression of vascular endothelial growth factor (VEGF) was firstly demonstrated in pterygia [10]. Pterygia present higher levels of VEGF compared with normal conjunctiva [11–16]. This brings about speculation that anti-VEGF drugs may be useful for pterygia patients. Bevacizumab is a recombinant human monoclonal antibody against VEGF, which is approved by FDA treating neoplasms. Many randomized controlled trials (RCTs) were performed to assess the safety and efficacy of bevacizumab in management of pterygium, showing conflicting conclusions [17–34]. A meta-analysis of 9 RCTs was conducted in 2014 [35], and the result showed that topical or subconjunctival bevacizumab had no statistically significant effect on preventing pterygium recurrence. However, the result was not been consistently supported by another 9 new RCTs published after 2014 [26–34]. The conclusion might be altered by the addition of 9 new studies. Therefore, we performed an additional meta-analysis to further evaluate the impact of bevacizumab on the recurrence and complication rates in the treatment of pterygium.

## 2. Methods

### 2.1. Search Strategy

The databases of Embase, PubMed, Web of Science, and the Cochrane Central Register of Controlled Trials (CENTRAL) were searched from their inception up until June 2017. Details of the search strategies were described in the Search Strategy file. Endnote software was used to exclude the duplications. Titles and abstracts were scrutinized to deduct apparently irrelevant studies. Full texts were retrieved and assessed for qualification. A manual search was executed by checking the reference lists of all retrieved studies and reviewing articles to distinguish studies not found by the electronic searches. Language was not restricted.

### 2.2. Inclusion and Exclusion Criteria

The articles were considered qualified if the studies fulfilled the following inclusion criteria: (1) participants: pterygium patients (including primary pterygium, impending recurrent pterygium, and recurrent pterygium); (2) intervention: topical or subconjunctival bevacizumab, regardless of operation or not. The dose of bevacizumab, follow-up periods, or length of fibrovascular growth passing the corneal limbus were not confined; (3) comparison: bevacizumab and control; (4) outcomes: recurrence and/or complication rates; and (5) publication type: RCT. RCTs without exact raw data available for extraction were excluded.

### 2.3. Outcome Measurements

The primary outcome measurements were recurrence and complication rates. Recurrence was diagnosed when any fibrovascular growth crossed the limbus and extended over the cornea to any distance by slit-lamp examination. The number of recurrences was estimated at the endpoint of the follow-up in each study. Complications such as lacrimation, inﬂammation, photophobia, conjunctival erythema, conjunctival ﬂap edema, conjunctival graft loss, subconjunctival hemorrhage, corneal dellen, severe conjunctival or corneal scarring, and systemic complications were counted. The number of complications at the last documenting time during the follow-up in each study was calculated.

### 2.4. Data Extraction

The data were extracted by two reviewers (Yi Sun and Bowen Zhang) independently. Discrepancies were resolved by discussion to reach a consensus between the investigators. The information collected from each study included the first author's last name, year of publication, study design, location and duration of the study, sample size including sex, age, and diagnoses, type of treatment and control, route of administration, and dose of bevacizumab.

### 2.5. Risk of Bias Assessment

Two reviewers (Yi Sun and Bowen Zhang) separately evaluated the risk of bias in each study according to the methods described in the Cochrane Handbook for Systematic Reviews of Interventions 5.3. The authors reviewed the studies and assigned a value of “high,” “low,” or “unclear” to the following items: (1) selection bias (Was there sufficient generation of the randomization sequence and allocation concealment?); (2) performance and detection bias (Was there blinding of participants, personnel, and outcome assessors?); (3) attrition bias (Were there incomplete outcome data and how to deal with this?); (4) reporting bias (Was there evidence of reporting outcome selectively?); and (5) other sources of bias (Were there any other potential threats to validity?). Any disagreement was discussed until a consensus was reached.

### 2.6. Statistical Analysis

The recurrence and complication rates were handled as dichotomous variables measured as the risk ratio (RR) with a 95% confidence interval (CI). Due to the diversity in sample size and the differences in clinical characteristics among the studies, it was presumed that heterogeneity existed even when no statistical significance was observed. Therefore, the data were pooled using a random effects model. Statistical heterogeneity among the studies was assessed by calculating a Cochran Q statistic and an *I* ^2^ statistic. Subgroup analysis and sensitivity analysis for the recurrence rate were carried out to evaluate the impact of the following factors on the results: (a) participants: primary pterygium, impending recurrent pterygium, and recurrent pterygium; (b) intervention: topical use or subconjunctival injection of bevacizumab; type of operation or not; and (c) follow-up periods: ≤6 months, 6～12 months, and ≥12 months. We explored asymmetry in funnel plots to detect publication biases. The analysis was performed using RevMan 5.3 (The Cochrane Collaboration, Copenhagen, Denmark).

## 3. Results

### 3.1. Literature Search

**Figure 1:**
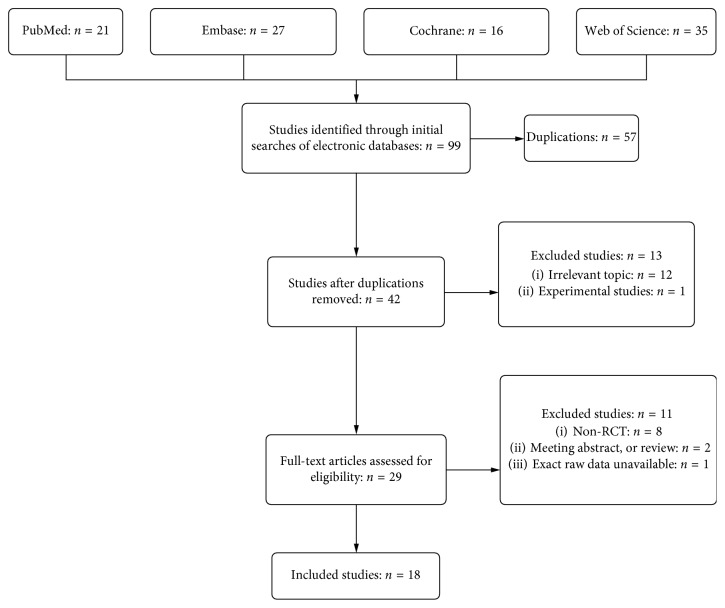
Flow diagram for the literature search and selection process.

Literature search and selection process are summarized in Figure 1. A total of 99 articles were initially enrolled. After removing duplications, the abstracts of the remaining studies were inspected, and 29 articles with possibly relevant trials were further identified in full texts. Eighteen randomized controlled trials (RCTs) were deemed eligible after a full text screening and were finally included in this meta-analysis.

### 3.2. Characteristics and Quality Assessment of the Included Studies

Characteristics of included studies are summarized in Table 1. In total, 18 RCTs were included in this review [17–34]. 17 studies were published in English and 1 in Chinese. 1045 eyes were enrolled: 561 in the bevacizumab group and 484 in the control group. Quality assessment was conducted according to Cochrane Handbook for Systematic Reviews of Interventions 5.3. The risks of biases in these studies are shown in supplementary data file (available here).

### 3.3. Meta-Analysis

**Figure 2:**
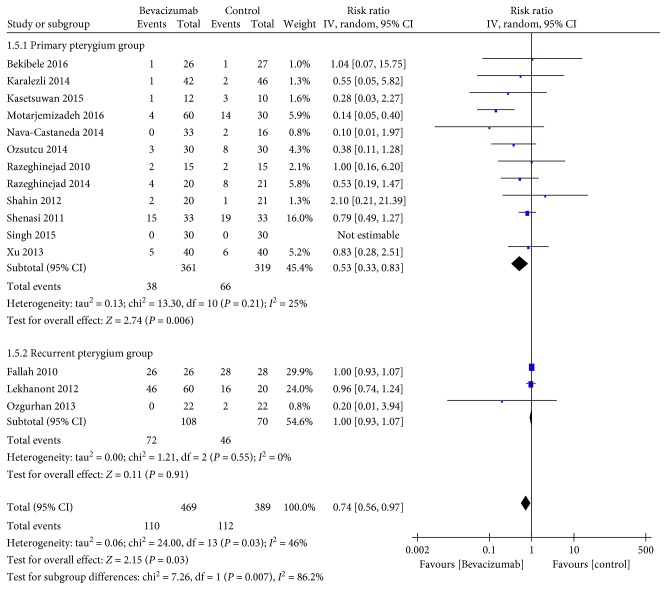
Subgroup analysis for the recurrence rates according to types of pterygium (*n* = 15, the remainder 3 studies without recurrence).

**Figure 3:**
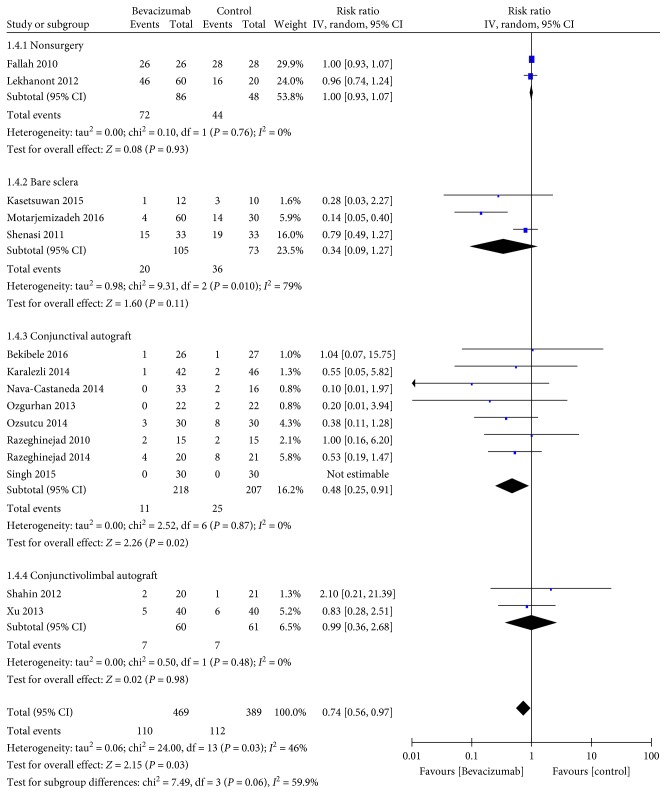
Subgroup analysis for the recurrence rates according to the treatment (*n* = 15, the remainder 3 studies without recurrence).

**Figure 4:**
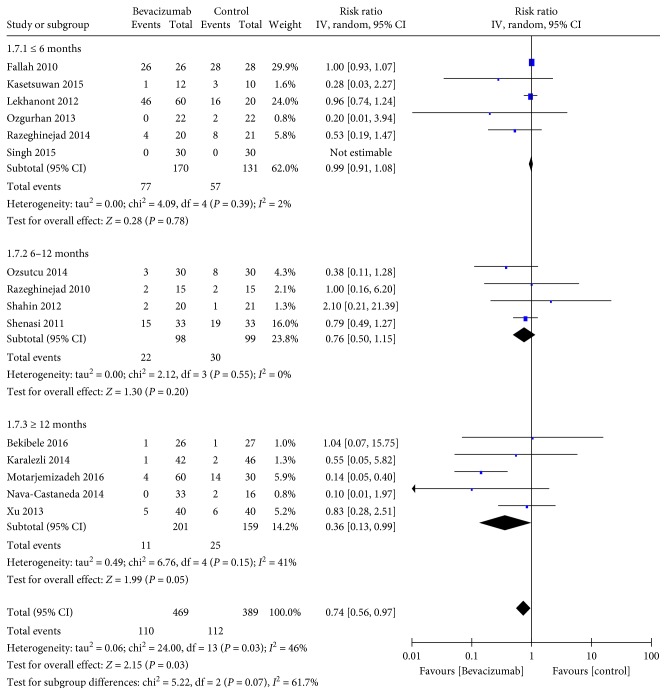
Subgroup analysis for the recurrence rates according to the follow-up time (*n* = 15, the remainder 3 studies without recurrence).

15 studies reported recurrences. Definitions of pterygium recurrence of the included randomized clinical trials are shown in Table 2. Overall recurrence rate of this meta-analysis was summarized in supplementary data file. The pooled results demonstrated that bevacizumab significantly reduced the pterygium recurrence (RR 0.74, 95% CI 0.56–0.97, *P*=0.03; *P*_heterogeneity_ = 0.03, *I* ^2^ = 46%). Subgroup analysis for the recurrence rate based on the pterygium types showed a statistically significant decrease in recurrence rate in the primary pterygium group (RR 0.53, 95% CI 0.33–0.83, *P*=0.006; *P*_heterogeneity_ = 0.21, *I* ^2^ = 25%), while not in the recurrent pterygium group (RR 1.00, 95% CI 0.93–1.07, *P*=0.91; *P*_heterogeneity_ = 0.55, *I* ^2^ = 0%) (Figure 2). Similarly, significant results in favor of bevacizumab were found in the conjunctival autograft group (RR 0.48, 95% CI 0.25–0.91, *P*=0.022; *P*_heterogeneity_ = 0.87, *I* ^2^ = 0%) (Figure 3) and the follow-up longer than 12 months group (RR 0.36, 95% CI 0.13–0.99, *P*=0.05; *P*_heterogeneity_ = 0.15, *I* ^2^ = 41%) (Figure 4). There was no statistically significant difference between the topical bevacizumab group (RR 0.38, 95% CI 0.12–1.23, *P*=0.11; *P*_heterogeneity_ = 0.002, *I* ^2^ = 76%) and the subconjunctival bevacizumab group (RR 0.87, 95% CI 0.70–1.07, *P*=0.18; *P*_heterogeneity_ = 0.64, *I* ^2^ = 0%) (supplementary data file).

17 studies reporting complications were analyzed. There was no statistically significant difference between bevacizumab group and control group (RR 0.87, 95% CI 0.66–1.13, *P*=0.30; *P*_heterogeneity_ = 0.52, *I* ^2^ = 0%) (supplementary data file). Further analysis of the subconjunctival hemorrhage rate showed that a statistically significant difference was not found between groups (RR 1.50, 95% CI 0.63–3.59, *P*=0.36; *P*_heterogeneity_ = 0.69, *I* ^2^ = 0%) (supplementary data file).

Publication bias for recurrence rates and complications was checked by evaluating funnel plots (supplementary data file).

## 4. Discussion

This meta-analysis, updated with 1045 eyes in 18 RCTs showed that bevacizumab would significantly reduce pterygium recurrence rate after surgery in either case of primary pterygium or use of conjunctival autograft or follow-up longer than 12 months. Complications of bevacizumab were not increased compared with the control.

An earlier meta-analysis performed by Hu indicated that bevacizumab had no statistically significant effect on preventing pterygium recurrence [35]. Hu included 9 RCTs, of which 7 reported recurrence and 8 reported complications, whereas in our current meta-analysis, we report raw data on recurrences in 15 and complications in 17 studies. The inclusion of more trials and more cases renders our analysis more statistically significant.

According to Prabhasawat [36], corneal recurrence with fibrovascular tissue covering the excision area and invading the cornea (grade 4) was the true recurrence. However, the definition of recurrence adopted in literatures varied. The inconsistent definition of pterygium recurrence in the included studies (Table 2) implied that the conclusion of the meta-analysis should be interpreted prudently. Study by Razeghinejad defined recurrence as any fibrovascular growth of conjunctival tissue extending more than 1.5 mm across the limbus [37]. In addition, data of recurrence in table 3 of the literature were found incorrect. Thus, the study was excluded. Moreover, the significant effect of bevacizumab on decreasing recurrence in the follow-up longer than 12 months group would suggest that longer follow-up in the future studies could further favor the effect.

RR for the overall recurrence rate was 0.74, with 95% CI [0.56, 0.97]. After removal of the study by Motarjemizadeh [31], *I* ^2^ decreased to 0% and RR was 0.98, with 95% CI [0.92, 1.05], but it did not affect the conclusive result in subgroup analysis on the pterygium type or administration route of bevacizumab. Therefore, sensitivity analysis was unstable and the heterogeneity was mainly caused by this study. However, there was no reason to exclude the study after comprehensive reading of the full text.

There was no statistically significant difference in overall complications and subconjunctival hemorrhage between bevacizumab group and control group, showing the safety of bevacizumab. The sensitivity analysis for the complication was stable. It is different from the previous meta-analysis by Hu [35], who reported the bevacizumab group was associated with a higher risk of developing subconjunctival hemorrhage.

The funnel plot for the recurrence and complication rates displayed asymmetry. This could be due to factors other than publication bias, including poor methodological quality, true heterogeneity, artefactual variation, and chance.

Our study had several potential limitations. First, the heterogeneity may result from different administration route of bevacizumab, different type of pterygium, surgeon's experience, and follow-up duration. Second, sensitivity analyses of the recurrence rate were not stable. Therefore, caution is required in their interpretation and more research is still needed.

Despite these limitations, the evidence from the updated meta-analysis shows that bevacizumab application following pterygium surgery provides a statistically significant decrease in recurrence rate in cases of primary pterygium, or use of conjunctival autograft, or follow-up longer than 12 months without an increase in complications. Further study of the long-term efficacy of bevacizumab on reducing pterygium recurrence based on the definition of true recurrence (grade 4) will be needed.

## Figures and Tables

**Table 1:** Characteristics of the included randomized clinical trials.

Author (year)	Location	No. of eyes (Bev/Con)	Administration route of bevacizumab	Mean age (Bev/Con, y)	Type of pterygium	Follow-up (m)	Treatment method
Fallah (2010)	Iran	26/28	Topical	49.96/51.61	Impending recurrent	3～6	Nonsurgery
Razeghinejad (2010)	Iran	15/15	Subconjunctival	45.8/41.6	Primary	8 vs 7.4	Conjunctival autograft
Banifatemi (2011)	Iran	22/22	Subconjunctival	41.95/44.13	Primary	1	Conjunctival autograft
Enkvetchakul (2011)	Thailand	34/40	Subconjunctival	51.5/49	Primary	6	Nonsurgery
Shenasi (2011)	Iran	33/33	Subconjunctival	58.67/55.94	Primary	9	Bare sclera
Shahin (2012)	Egypt	20/21	Subconjunctival	58.40/57.58	Primary	8	Conjunctivolimbal autograft
Lekhanont (2012)	Thailand	60/20	Subconjunctival	48.98/48.27	Impending recurrent	3	Nonsurgery
Ozgurhan (2013)	Turkey	22/22	Topical	48.4/50.5	Recurrent	6	Conjunctival autograft
Xu (2013)	China	40/40	Subconjunctival	44/41	Primary	12	Conjunctivolimbal autograft
Nava-Castaneda, A (2014)	Mexico	33/16	Subconjunctival	48.75/47.8	Primary	12	Conjunctival autograft
Karalezli (2014)	Turkey	42/46	Topical	58.82/53.04	Primary	29.3 VS 28.5	Conjunctival autograft
Razeghinejad(2014)	Iran	20/21	Subconjunctival	41.95/44.13	Primary	6	Conjunctival autograft
Ozsutcu(2014)	Turkey	30/30	Subconjunctival	43.25/41.68	Primary	9	Conjunctival autograft
Kasetsuwan(2015)	Thailand	12/10	Topical	50.7/59.3	Primary	3	Bare sclera
Hwang(2015)	Korea	36/33	Topical	71.3/73.4	Primary	6	Bare sclera
Singh(2015)	India	30/30	Subconjunctival	37.33	Primary	3	Conjunctival autograft
Bekibele(2016)	Nigeria	26/27	Subconjunctival	49.2/52.0	Primary	18.35	Conjunctiva autograft
Motarjemizadeh(2016)	Iran	60/30	Topical	39.47/40.97	Primary	12	Bare sclera

Bev: bevacizumab; Con, control; y, year; m, month.

**Table 2:** Definition of pterygium recurrence of the included randomized clinical trials.

Author (year)	Definition of recurrence
Fallah (2010)	Fibrovascular tissue stretching onto cornea
Razeghinejad (2010)	Fibrovascular tissue extending more than 1.5 mm across limbus
Shenasi (2011)	Fibrovascular growth crossing limbus and extending over the cornea to any distance
Shahin (2012)	4 grades classified
Lekhanont (2012)	Fibrovascular tissue invading cornea or when the lesion was categorized as grade 4
Ozgurhan (2013)	No specific definition
Xu (2013)	Fibrovascular tissue invading cornea
Nava-Castaneda, A (2014)	4 grades classified
Karalezli (2014)	Fibrovascular growth passing the corneal limbus by more than 1mm
Razeghinejad (2014)	More than 1.5 mm of fibrovascular tissue overgrowth on cornea and any fibrovascular tissue crossing limbus
Ozsutcu (2014)	Any fibrovascular growth of conjunctival tissue extending more than 1.5 mm across limbus
Kasetsuwan (2015)	4 grades classified
Singh (2015)	4 grades classified
Bekibele (2016)	Growth of fibrovascular tissue 1 mm or more into cornea
Motarjemizadeh (2016)	New vessels or fibrovascular connective tissues crossing corneal limbus

## Supplementary Materials

Supplementary Materials The supplemental data file mainly described risk of bias assessment, the overall effect of Bevacizumab on reducing recurrence rates, the insignificant difference of recurrence rates between topical and subconjunctival bevacizumab, the complications without increase, and the publication bias.
