# Supplementary material for: Efficacy and Safety of Bevacizumab in the Treatment of Pterygium: An Updated Meta-Analysis of Randomized Controlled Trials
Source: J Ophthalmol. 2018 Sep 5;2018:4598173. doi: 10.1155/2018/4598173 (PMC6145151; doi:10.1155/2018/4598173)
Supplement: Supplementary Materials — The supplemental data file mainly described risk of bias assessment, the overall effect of Bevacizumab on reducing recurrence rates, the insignificant difference of recurrence rates between topical and subconjunctival bevacizumab, the complications without increase, and the publication bias. [file 4598173.f1.doc]

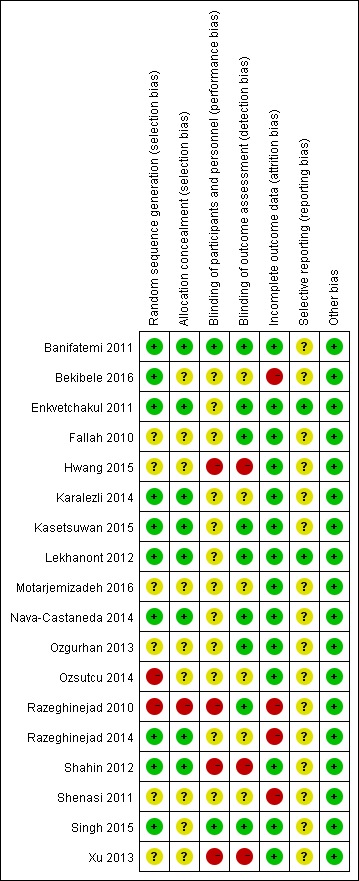


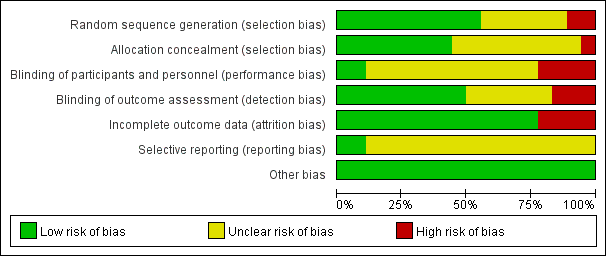


Figure 1. Risk of bias assessment of RCTs.


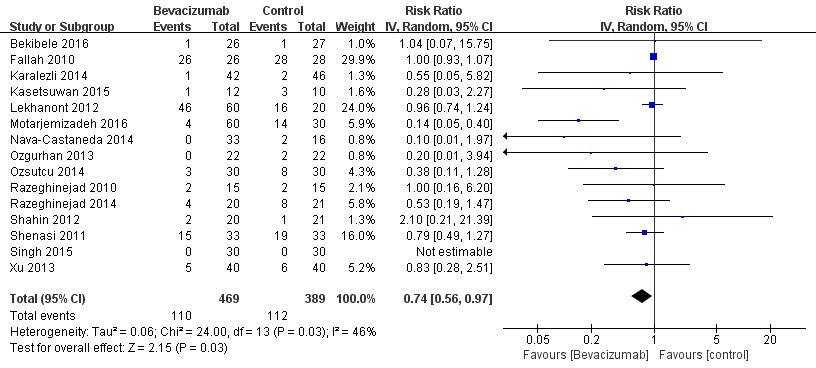


Figure 2. Forest plot for the overall recurrence rates of pterygium (n=15, the remainder 3 studies without recurrence).


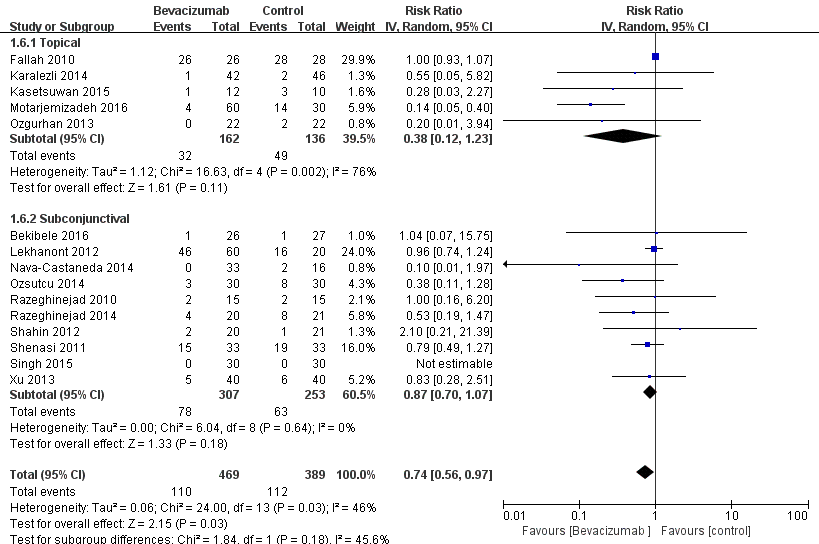


Figure 3. Subgroup analysis for the recurrence rates according to the different administration of bevacizumab(n=15, the remainder 3 studies without recurrence).

A


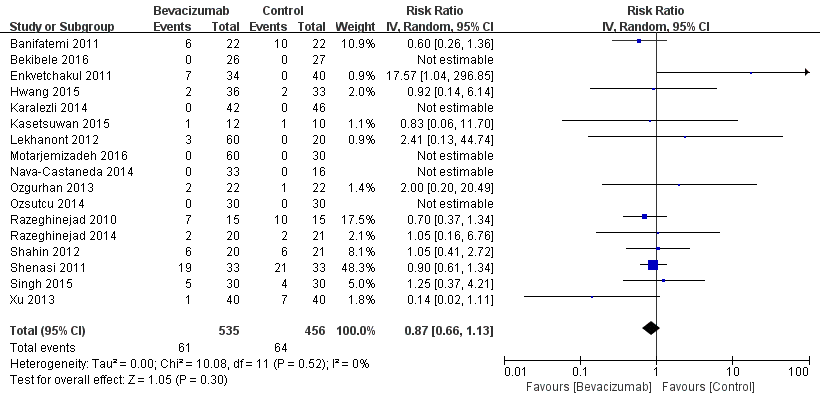


B


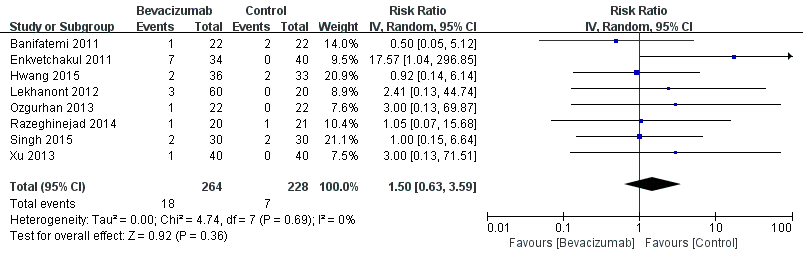


Figure 4. Forest plot for the overall complications (A, n=17) and subconjunctival hemorrhage (B, n=8).


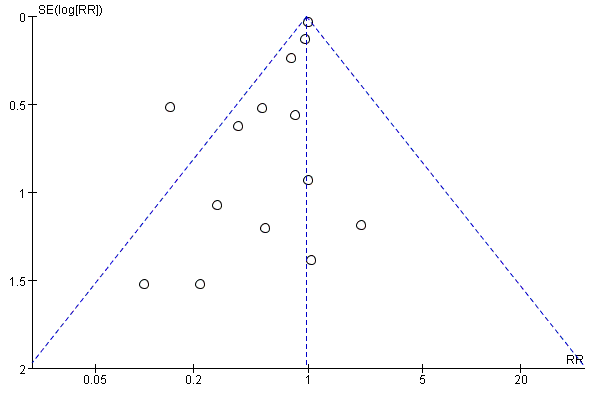


A


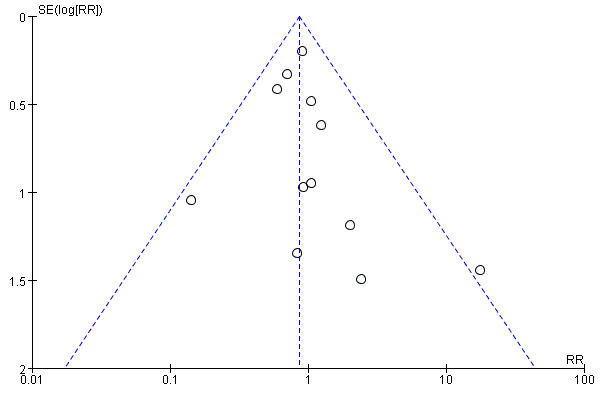


B

Figure 5. Funnel plot showing the publication bias for the recurrence rate (A) and complications (B).
